# Identifying the best candidate for focal therapy: a comprehensive review

**DOI:** 10.1038/s41391-024-00907-y

**Published:** 2024-10-23

**Authors:** Alireza Ghoreifi, Leonard Gomella, Jim C. Hu, Badrinath Konety, Luca Lunelli, Ardeshir R. Rastinehad, Georg Salomon, Samir Taneja, Rafael Tourinho-Barbosa, Amir H. Lebastchi

**Affiliations:** 1 Department of Urology, University of Southern California, Los Angeles, CA USA; 2 Department of Urology, Thomas Jefferson University, Philadelphia, PA USA; 3 Department of Urology, Weill Cornell Medicine, New York-Presbyterian Hospital, New York, NY USA; 4 Allina Health Cancer Institute, Minneapolis, Minneapolis, MN USA; 5 Department of Urology, Hospital Louis Pasteur, Chartres, France; 6 The Smith Institute for Urology at Lenox Hill, New York, NY USA; 7 Martini Clinic, Prostate Cancer Center Hamburg-Eppendorf, Hamburg, Germany; 8 Department of Urology, NYU Langone Health, New York, NY USA; 9 Department of Urology, Institut Mutualiste Montsouris, Université Paris-Descartes, Paris, France

**Keywords:** Prostate cancer, Prostate cancer

## Abstract

**Background:**

Despite the evidence supporting the use of focal therapy (FT) in patients with localized prostate cancer (PCa), considerable variability exists in the patient selection criteria across current studies. This study aims to review the most recent evidence concerning the optimal approach to patient selection for FT in PCa.

**Methods:**

PubMed database was systematically queried for studies reporting patient selection criteria in FT for PCa before December 31, 2023. After excluding non-relevant articles and a quality assessment, data were extracted, and results were described qualitatively.

**Results:**

There is no level I evidence regarding the best patient selection approach for FT in patients with PCa. Current international multidisciplinary consensus statements recommend multiparametric magnetic resonance imaging (mpMRI) followed by MRI-targeted and systematic biopsy for all candidates. FT may be considered in clinically localized, intermediate risk (Gleason 3 + 4 and 4 + 3), and preferably unifocal disease. Patients should have an acceptable life expectancy. Those with prostate volume >50 ml and erectile dysfunction should not be excluded from FT. Prostate-specific antigen (PSA) level of < 20 (ideally < 10) ng/mL is recommended. However, the utility of other molecular and genomic biomarkers in patient selection for FT remains unknown.

**Conclusions:**

FT may be considered in well-selected patients with localized PCa. This review provides a comprehensive insight regarding the optimal approach for patient selection in FT.

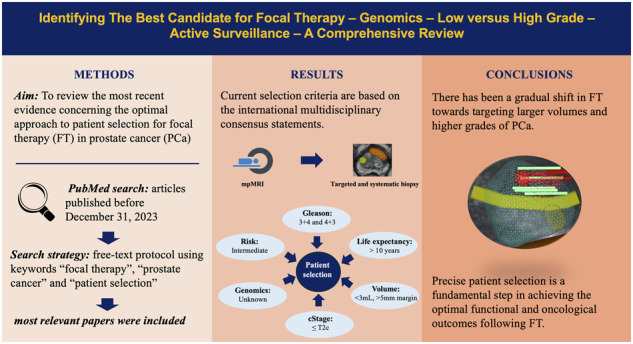

## Background

Focal therapy (FT) has emerged as a viable treatment option in the management of patients with localized prostate cancer (PCa). The goal of FT is to mitigate the side effects commonly associated with more aggressive treatments without compromising cancer control [1–3]. Numerous FT modalities are currently available, including high intensity focused ultrasound (HIFU), cryotherapy, irreversible electroporation (IRE), laser, photodynamic therapy (PDT), and brachytherapy (BT). Several single-center and multi-institutional studies have reported the outcomes of these novel therapies [4]. Currently, the American Urological Association (AUA) guidelines consider FT in select, appropriately informed patients with intermediate-risk PCa, with an emphasis on prioritizing enrollment in clinical trials [5]. However, patients should be informed that high-quality data comparing the outcomes of FT to other PCa management options, including radiation therapy, surgery, and active surveillance, are currently lacking.

**Fig. 1:**
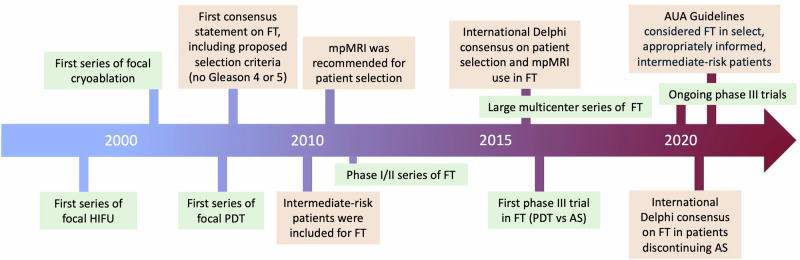
The evolution of focal therapy and patient selection criteria over the past two decades. HIFU high-intensity focused ultrasound, PDT photodynamic therapy, mpMRI multiparametric magnetic resonance imaging, FT focal therapy, AS active surveillance, AUA American Urological Association.

Despite the evidence supporting the use of FT in patients with localized PCa, reported studies exhibit considerable variability in terms of patient selection criteria and treatment planning approaches [4]. Precise selection of patients is a crucial step in achieving the optimal outcomes following FT. The identification of the best candidate has evolved dynamically in the past two decades alongside the increasing comprehension and constraints of FT (Fig. 1). Nevertheless, this process is still dependent on the current available evidence, expert opinions, and international multidisciplinary consensus statements [6–13]. These statements incorporate several criteria, including the type of imaging, prostate biopsy techniques, pathological features and anatomical location of the lesion(s), and a comprehensive assessment of the patient’s overall health and life expectancy. In recent years, advancements in diagnostic modalities, such as multiparametric magnetic resonance imaging (mpMRI) have reshaped the paradigm for patient selection [14]. In addition, the emergence of novel genomic markers has engendered optimism regarding the prospect of refining risk stratification for patients with PCa [15].

The objective of this study is to review the most recent evidence concerning the optimal approach to patient selection for FT in PCa.

## Methods

**Fig. 2:**
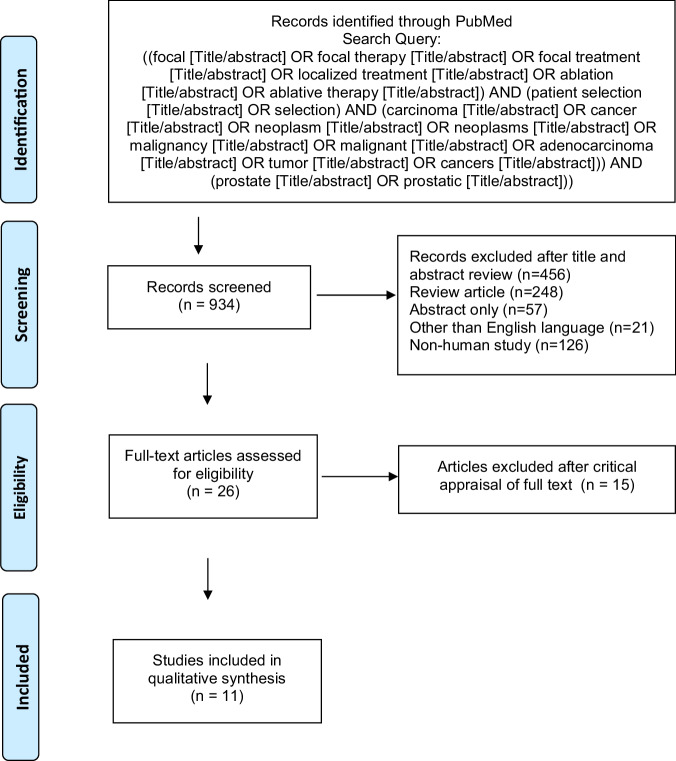
The selection process of the articles assessing patient selection in focal therapy for prostate cancer.

Our search was performed using PubMed database for articles published before December 31, 2023. A systematic review was conducted according to the Preferred Reporting Items for Systematic Review and Meta-analyses (PRISMA) statement to assess patient selection criteria in FT for PCa. Unrelated articles, letters, editorial comments, replies from authors, non-human and non-English language articles were excluded. The detailed search terms, filters, and exclusions are presented in Fig. 2.

After critical and quality assessments, data were extracted, and results were described qualitatively. Two investigators (A.G. and A.H.L.) were independently involved in the data assessment and extraction. The following data were extracted: first-author, year of publication, consensus methos, consensus threshold, number of expert participants, response rates, characteristics of participants, summary of recommendations for imaging and biopsy in FT, and summary of consensus statements in patient selection for FT.

## Results

Several single-center and multicenter reports on FT with different sources of energy have been reported during the past two decades, with the majority being small-sample size single-arm phase I or II studies [16–23]. With increasing experience in this field, multicenter reports and larger sample size studies have been recently published [24–28]. The only randomized trial reported to date in the field of FT included patients with low-risk PCa who underwent focal PDT [29]. Hence, due to the lack of level I evidence, patient selection for FT is currently based on several international multidisciplinary consensus statements [6–13]. These statements define several aspects of the ideal patient selection for FT in PCa, including diagnostic modalities (imaging and biopsy) to characterize the lesion(s), as well as patient-related and disease-related factors contributing to the optimal decision making.

In this systematic review, a total of 11 articles were included in the qualitative synthesis. Characteristics of these studies are presented in Table 1. Additionally, a summary of recommendations for imaging and biopsy, along with patient selection criteria outlined in these reports, is provided in Tables 2 and 3.

**Table 1:** Characteristics of the studies assessing patient selection in focal therapy for prostate cancer.

Study, year (ref)	Consensus method (threshold)	Number of participants and response rates	Characteristics of the participants
**Eggener et al**. [6]	NA	14 experts were included in the paper, but the total no of contributors was not mentioned.	International multidisciplinary group with expertise in prostate cancer (surgeons, radiotherapists, medical oncologists, radiologists, pathologists, and epidemiologists)
**de la Rosette et al**. [7]	3-stage informal consensus process during an in-person meeting (NA)	22 experts	15 urologists, 3 radiologists, 3 radiation oncologists, and 1 pathologist
**Ahmed et al**. [32]	In person break-out sessions followed by a group agreement (NA)	46 experts	17 urologists, 13 radiologists, 3 radiation oncologists, 1 oncologist, 1 pathologist, 1 biostatistician, and 10 other physicians and scientists
**Muller et al**. [31]	3-stage informal consensus process during an in-person meeting (NA)	16 experts	9 urologists, 5 radiologists, and 2 basic researchers
**van den Bos et al**. [8]	4-stage Delphi; 3 online and 1 in person (NA)	48 experts. The response rates for the questionnaires were 88%, 85%, and 96% in rounds 1, 2, and 3, respectively.	35 urologists, 6 radiologists, 2 radiation oncologist, 2 pathologists, 2 surgeons, and 1 from surgery and interventional science
**Donaldson et al**. [9]	Modified 2-stage RAND/UCLA appropriateness method followed by an in-person meeting (IPRAS score >0)	15 voting members, 1 independent chairperson with expertise in consensus methodology, and 4 nonvoting observers.	Among 15 voting members, 13 were urologists and 2 oncologists
**Scheltema et al**. [14]	4-stage Delphi; 3 online and 1 in person (agreement >80%)	90 out of 166 (54%) accepted the invitation, and the response rate was 100% (90/90), 94% (85/90), 88% (79/90) for rounds 1, 2, and 3, respectively.	Among 78 experts who completed the 3 rounds, 72% were urologists, 16% radiologists, 3% pathologists, 3% radiation oncologists and 6% scientists.
**Tay et al**. [10]	4-stage Delphi; 3 online followed by 1 in person (agreement >80%)	First round: 51 out of 113 (45%); Second and third rounds: response rate 92.1% (47 in each round); Fourth round: 16 experts, all of whom had completed three online rounds.	70% urologists, 11% radiologists, 9% physicist/researchers, 4% radiation oncologists, 2% medical oncologists, 2% pathologists, 2% interventional urologic oncologists.
**van Luijtelaar et al**. [11]	4-stage Delphi method (NA)	37 out of 75 (49%) accepted the invitation. Response rates were 100% (37/37), 70% (26/37), 68% (25/37), and 65% (24/37) for rounds 1 to 4, respectively.	19 (51%) urologists, 14 (38%) (interventional) radiologists, 1 (3%) radiation oncologist, 1 (3%) researcher, 1 (3%) technical physician, and 1 (3%) engineer.
**Tan et al**. [13]	4-stage Delphi; 3 online followed by 1 in person (agreement ≥ 80%)	56 out of 91 (61%) filled out the initial survey. Response rates for the second and third rounds were 100% (56/56) and 88% (49/56), respectively. A total of 17 panelists attended the face-to-face meeting.	84% urologists, 14% radiologists, 2% radiation oncologist.
**Borkowetz et al**. [12]	Group consensus (strong consent >95%, consent 75–95%, major consent 50–75%, dissent < 50%)	18 German experts	Urologists, radio-oncologists, radiologist, and pathologist.

*UCLA* University of California Los Angeles, *IPRAS* Inter-percentile range adjusted for symmetry, *NA* not available.

**Table 2:** Summary of recommendations for imaging and biopsy in focal therapy.

**de la Rosette et al**. [7]
• Candidates for FT should ideally undergo transperineal template-mapping biopsies, although a state-of-the-art multifunctional MRI with TRUS biopsy at expert centers may be acceptable
**Ahmed et al**. [32]
• mpMRI and transperineal prostate mapping biopsy can improve PCa care and risk stratification before FT.
**Muller et al**. [31]
• mpMRI is the optimum approach to achieve the objectives needed for FT, if made on a high-quality machine (3 T with/without endorectal coil or 1.5 T with endorectal coil) and judged by an experienced radiologist. • Structured and standardized reporting of prostate MRI is paramount. • State of the art mpMRI is capable of localizing small tumors for focal therapy. • State of the art mpMRI is the technique of choice for follow-up of focal ablation.
**Donaldson et al**. [9]
• MRI-targeted or template-mapping biopsy should be used to plan treatment.
**Scheltema et al**. [14]
• mpMRI should be performed in patients with prior negative biopsies if clinical suspicion remains. • mpMRI should not be performed as stand-alone diagnostic tool or with mpMRI-targeted biopsies only. • mpMRI should be performed following standard biopsy-based PCa diagnosis in both the planning and follow-up of FT. • MRI-TRUS fusion is the recommended technique to perform biopsies following mpMRI. • Systematic biopsies are still required for FT planning in biopsy-naïve patients and patients with residual PCa after FT. • Repeat biopsies should be taken during the follow-up of FT. • The final decision to perform FT should be based on histopathology and not be based on mpMRI results alone. • Only in expert centers, where the quality is assured and own results are monitored, mpMRI may be performed in all patients suspected of PCa. • Only in expert centers, deferral of repeat biopsy may be considered in case of a negative mpMRI. • It should be our goal to guarantee high-quality mpMRI throughout the urological community before implementing it as standard of care.
**Tay et al**. [10]
▪ mpMRI is a standard imaging tool to select patients for FT. • mpMRI is essential particularly in the setting of targeted/lesional ablation. • mpMRI is preferred whenever possible when FT is planned (core group) ▪ In the presence of an mpMRI-suspicious lesion (PIRADSv2 4/5), histological confirmation is necessary prior to treatment with FT. ▪ MRI–TRUS fusion biopsy is adequate in assessing an mpMRI lesion prior to FT. • VET/cognitive fusion biopsy can be considered adequate in expert hands (core group) ▪ Systematic biopsies remain necessary to assess mpMRI-negative areas prior to treating a histologically confirmed mpMRI lesion. ▪ Where mpMRI is unavailable or contraindicated, 12 core TRUS biopsy alone is insufficient for patient selection for FT.
**van Luijtelaar et al**. [11]
• Patients who require targeted ablation of specific focus with in-bore transperineal or transrectal technique using mpMRI as the standard imaging tool. • Will have systematic biopsies as necessary.
**Tan et al**. [13]
• mpMRI/US-guided fusion biopsy and a 12-core systematic biopsy is recommended for men on active surveillance prior to considering focal therapy. • If unable to undergo mpMRI, patients will require a 3D mapping biopsy of the prostate to determine if they are a candidate for focal therapy. • No metastatic workup is usually required prior to considering focal therapy
**Borkowetz et al**. [12]
▪ Patients considering FT should undergo mpMRI, mpMRI fusion biopsy, and systematic biopsy. ▪ If MRI fusion biopsy is not possible, a template-based biopsy may be considered to be performed as an alternative.

*FT* Focal therapy, *mpMRI* Multiparametric magnetic resonance imaging, *TRUS* transrectal ultrasound, *PCa* Prostate cancer, *PIRADS* Prostate Imaging Reporting & Data System.

**Table 3:** Summary of consensus statements in patient selection for focal therapy.

	Patient factors	Disease factors	Biomarkers
**Eggener et al**. [6]	-	- Clinical stage T1 or T2a - No Gleason 4 or 5 - Maximum 20% cancer in each core - Maximum 7 mm of cancer in each core - Maximum 33% of total cores with cancer - Single lesion with a maximum size of 12 mm in imaging - Maximum 10 mm length of capsular contact - No evidence of extraprostatic extension or seminal vesicle invasion	- PSA < 10 ng/ml - PSA density < 0.15 ng/ml/ml - PSA velocity < 2 ng/ml yearly in the year prior to diagnosis
**de la Rosette et al**. [7]	- Life expectancy of ≥ 10 years - Patients with previous prostate surgery or lower urinary tract symptoms should be counseled with caution. - No previous radiotherapy to the prostate or pelvis	- Low to moderate risk cancer. - Clinical T2a or less N0M0 - Radiologic ≤ T2b N0M0 disease. - Apical or anterior lesions may be technically difficult to manage with existing treatment modalities.	-
**van den Bos et al**. [8]	- Life expectancy >10 years	- Clinical stage, T1c–T2a - Gleason score 3 + 3 or 3 + 4 - Prostate volume, any; except in case of HIFU, < 40 ml - No previous treatment of the primary prostate cancer, hormone treatment within the past 6 months before trial, radiation to the pelvis, or active urinary tract infection - No extracapsular extension, seminal vesicle invasion, lymph node or bony metastasis	- PSA < 15 ng/ml - PSA >15 ng/ml should be counseled with caution.
**Donaldson et al**. [9]	-	- Intermediate risk PCa - Prostate volume or age should not be a primary determinant of eligibility. - Foci of indolent cancer can be left untreated when treating the dominant index lesion.	-
**Tay et al**. [10]	- Life expectancy considerations are similar to those stated in major guidelines - No upper or lower age boundary - Lack of erectile function and mild to moderate lower urinary tract symptoms should not exclude a patient from FT. - Prostate volumes < 50 ml.	- Low- and intermediate-risk - Clinically localized cancer, with a single favorable lesion/size. - Gleason 3 + 4 (ideal) or 4 + 3 (acceptable) cancer - Cancer foci < 1.5 ml on mpMRI - Foci < 3 ml but localized to one hemi-gland are suitable (depending on gland volume and energy source) - Cancer foci occupying 20% of the prostate on mpMRI - Foci up to 25% but localized to one hemi-gland	- PSA⩽10 ng/ml
**van Luijtelaar et al**. [11]	- Patients with desire to preserve erectile or sphincter function.	- Intermediate risk cancer (Gleason score 7, cStage T2b) - MRI-visible local recurrence - de novo cancer with Gleason ≤ 4 + 3 - ≤ 10–15 mL tumor	- PSA 10–20
**Tan et al**. [13]	- Age 60 − 80 when coming off active surveillance.	- Gleason 3 + 4 cancer with localized disease. - No multi-focal grade group ≥ 2 lesions	- PSA < 10 ng/ml
**Borkowetz et al**. [12]	- Unsuspected digital rectal examination	- Unilateral, localized low-risk PCa - Gleason score 6 - Maximum 50% positive biopsy cores of only one lobe	- PSA < 10 ng/ml

*PSA* Prostate-specific antigen, *mpMRI* multiparametric magnetic resonance imaging, *FT* Focal therapy, *PCa* Prostate cancer.

### Lesion characterization

The first step in assessing the eligibility of a patient who might be eligible for FT is to characterize the PCa lesion(s). FT initially relied on the cancer characteristics of transrectal ultrasound (TRUS)-guided biopsies [30]. The introduction of mpMRI has revolutionized cancer detection and consequently improved the process of patient selection for FT. The use of mpMRI in FT candidates was first presented in a consensus statement by the experts in this field in 2010 [7]. Subsequently, panels of experts re-emphasized the importance of mpMRI as the preferred approach for accomplishing the necessary objectives in FT [31, 32]. Recent consensus panels on patient selection for FT have consensually concurred that mpMRI stands as the preferred imaging modality for preoperative evaluation [7–13]. mpMRI has a high sensitivity in detecting clinically significant PCa. In a Cochrane meta-analysis comparing MRI to template biopsies in biopsy-naïve and repeat-biopsy settings, MRI had a pooled sensitivity and specificity of 0.91 and 0.37 for the International Society of Urological Pathology (ISUP) grade >2 cancers, respectively. For grade >3 cancers, the pooled sensitivity of MRI was 0.95, and the specificity was 0.35 [33].

MRI-TRUS fusion is the recommended technique to perform biopsies following mpMRI. The findings of PRECISION trial (PRostate Evaluation for Clinically Important Disease: Sampling Using Image-guidance Or Not?) demonstrated that an mpMRI-guided biopsy leads to higher detection of clinically significant PCa while avoiding detection of insignificant disease [34]. According to an International Delphi Consensus, in the presence of an mpMRI-suspicious lesion (i.e., Prostate Imaging Reporting & Data System: PIRADS 4 or 5), histological confirmation using MRI-TRUS-fusion biopsy is necessary prior to treatment with FT [10]. Systematic biopsy is still required in this setting to assess mpMRI-negative areas prior to treating a histologically confirmed mpMRI lesion. However, minimum standard for the extent of systematic biopsy outside of the mpMRI lesion (i.e., number of cores/approach) remains indeterminate. When mpMRI is unavailable, three-dimensional (3D) mapping biopsies are recommended [13]. Prostate-specific membrane antigen (PSMA) positron emission tomography (PET) scan, an effective imaging modality in patients with PCa, may be used more frequently in the future as experience increases with its use in the field of FT [35].

Prostate biopsy can be performed using the transrectal (TR) or transperineal (TP) approach. The TR approach has been traditionally favored due to its less invasive nature and feasibility under local anesthesia [36]. However, a significant drawback of this approach is its relatively high rate of infectious complications. In a systematic review of 165 articles, TR, compared to TP approach, was associated with a significantly higher incidence of sepsis (0.8% vs 0.1%) and hospitalization (1.1% vs. 0.9%) [37]. Recent prospective studies have demonstrated that the TP approach can safely omit antibiotics without increasing the risk of infection, while maintaining comparable detection rates of PCa to the TR approach [38–41]. Consequently, the TP approach is now preferred for patients undergoing prostate biopsy [36].

### Patient features

Overall health and clinical features of the patients are important points that should be considered during appropriate candidate selection for FT. According to an the Delphi Consensus by Tay et al., life expectancy considerations are similar to those stated in major guidelines, with no upper or lower boundary beyond which FT is contraindicated [10]. According to the current guidelines, minimum estimated life expectancy of 8–10 years is required in order for treatment to result in a reduction in the risk of death [5]. Nevertheless, Tan et al. considered the age range of 60–80 when considering FT for patients who are discontinuing active surveillance [13]. Additionally, similar to other surgical procedures, those with less comorbidities are more appropriate candidates for FT compared to sicker patients.

Genitourinary symptoms are also important when considering a patient for FT. Although preservation of erectile function is an important reason for choosing FT over radical treatments, the lack of erectile function at baseline should not exclude a patient from FT [10]. In addition, the presence of mild to moderate lower urinary tract symptoms are not contraindications for FT. Men with prostate volumes of less than 50 ml are more suitable for FT compared to those with prostate volumes >50 ml. Patients with a larger prostate should not be excluded from FT but they need to be counseled with caution. FT in these patients depends on the location of index lesion, amount of tissue requiring ablation, and type of ablative energy [10]. For instance, in the case of HIFU treatment for a large prostate, ultrasound waves may dissipate over longer focal points, resulting in prostatic edema. This could potentially displace the treatment target from the firing zone, especially in lesions located in the anterior zone [42]. On the contrary, FT of posterior lesions is not affected by the prostate size.

Initial consensus statements for FT patient selection have excluded salvage cases, including those with previous treatment of the primary cancer within the prostate, recent hormone treatment for PCa, and previous radiation to the pelvis [8]. In recent years, more experience has been gained with salvage FT. The recent AUA guidelines recommend offering cryoablation and HIFU to patients with biopsy-documented PCa recurrence after primary radiation as part of a shared decision-making approach [43]. Nevertheless, data regarding salvage FT following primary PCa ablation is limited. Salvage FT should preferably be performed in experienced centers as part of a clinical trial or well-designed prospective cohort study. In addition, patients should be made aware of the potential complications and functional outcomes associated with this procedure [44–46].

Finally, patients should understand the lack of randomized clinical trial and long-term outcomes following FT. In addition, due to the slight risk of infield recurrence resulting from incomplete ablation or outfield recurrence caused by small, overlooked satellite lesions, or the de novo occurrence of PCa in the untreated gland, the patient must be compliant for close surveillance after treatment [47]. Patients may require re-treatments (e.g., repeat FT, radiation, or radical prostatectomy), which could lead to suboptimal outcomes when compared to primary treatments [44–46].

### Disease features

#### Pathological characteristics

Gleason grade is an important factor to be considered in the evaluation of a patient with PCa for FT. In the past decade, there has been a shift from low-grade cancers toward a higher grade. In the earlier days, FT was only considered for low-risk patients, and the presence of Gleason 4 in the biopsy was among the exclusion criteria. In 2010, the consensus statement by de la Rosette et al. was first to include patients with Gleason pattern 4 PCa for FT [7]. The advancement of imaging and biopsy techniques has led to wide acceptance of these new criteria among focal therapists. Currently, major guidelines recommend active surveillance as the preferred management option for patients with low-risk (i.e., Gleason 3 + 3) PCa [5, 48]. This recommendation is based on the high-level evidence from the ProtecT trial, which demonstrated no significant differences in long-term all-cause mortality among patients with localized PCa who underwent radical prostatectomy, radiation therapy, or active monitoring [49]. FT is currently accepted for patients with intermediate-risk PCa, and those having Gleason 3 + 4 cancer representing the ideal candidates [5, 10]. Given the limited evidence supporting the use of FT in patients with Gleason grade >7, this treatment should be offered with caution and only to those in whom additional diagnostic evaluations have confirmed the absence of extraprostatic disease (Expert Opinion). In cases where a patient presents with a single core of Gleason 8, accompanied by multiple cores of Gleason 6 and 7 in surrounding areas, the single Gleason 8 core may disproportionately represent the disease burden. In such cases, whole mount pathology may reveal the predominant presence of Gleason ≤ 7 following gland extirpation. It is important to note that various consensus statements have employed different criteria for calculating PCa risk groups (e.g., D’Amico vs. National Comprehensive Cancer Network: NCCN). However, this discrepancy has minimal impact on the selection criteria for FT.

#### Anatomical characteristics

Patients with PCa who are candidates for FT should have a clinically localized (clinical stage ≤ T2c) disease [10, 13]. While select patients with extra-prostatic extension (T3a) may be considered for FT, those with seminal vesicle invasion (T3b) and bladder neck invasion (T3b) should be counseled with caution [24, 25]. FT in such a high-risk group of patients should only be performed by highly experienced urologists. It is worth noting that FT in patients with T3 disease may be associated with a higher failure rate. In a prospective study of 625 consecutive patients with non-metastatic clinically significant PCa undergoing focal HIFU stage T3 was a significant predictor of failure, with multivariable hazard ratio of 3.06 (95%CI 1.11–8.44; *p* = 0.03) [24].

The tumor volume in both imaging and needle biopsies was a limitation to FT during the period when random biopsies were used for the detection of PCa. In the initial consensus statement for FT by Eggener et al., the inclusion criteria consisted of a single lesion with a maximum size of 12 mm in the imaging, as well as maximal cancer percentage in core < 20%, maximal cancer length in each core < 7 mm, and maximal cores with cancer < 33% [6]. Nowadays, with the availability of mpMRI, evaluation of cancer size is calculated more accurately. According to recent consensus statements, visible cancer foci < 1.5 ml on mpMRI are suitable for FT. Furthermore, foci < 3 ml but localized to one hemi-gland are also be considered for FT if an appropriate ablation with a good margin (5–10 mm) can be achieved. It is important to highlight that optimizing the treatment margin in FT is crucial. An excessive treatment margin might harm critical structures, while insufficient margins could compromise the treatment outcomes. The type of energy source plays an important role when making decisions for FT of lesions of >1.5 mL. Considering the prostate volume, cancer foci occupying 20% of the prostate on mpMRI are deemed suitable for FT. Additionally, foci occupying up to 25%, yet confined to one hemi-gland, may also be considered for FT [10].

The location of PCa lesion is not a limitation for FT but may impact the choice of energy source used for ablation. Prostatic edema during HIFU may push away the target area and decrease the efficacy of treatment, particularly for anterior lesions [42]. Consideration should also be given to apical lesions. Due to the proximity of these lesions to the sphincter, thermal-based FT modalities, such as cryotherapy and HIFU can cause some degree of sphincteric dysfunction, which may result in a higher rates of urinary incontinence [42].

Patients with multi-focal lesions are not ideal candidates for FT [13]. Most of the experience on FT has specifically included unilateral and unifocal lesions. Although feasible, ablation of multifocal tumors may attenuate the advantages of FT and compromised the oncological control associated with whole-gland treatments. However, select cases with multifocal disease may be considered for FT in experienced hands.

#### Molecular biomarkers

Prostate-specific antigen (PSA) is a known biomarker used in patient selection for FT. Most consensus statements agree on the PSA level of < 10 ng/mL when considering a patient for FT [6, 10, 12, 13]. However, some experts suggest that a PSA level ranging between 10 and 20 ng/mL should also be considered acceptable [11]. It should be noted that decision about performing FT should not be based solely on PSA levels, particularly when those levels exhibit volatility and may be influenced by other factors, such as infections or urinary obstruction. Therefore, even those with elevated PSA levels above 20, may be considered for FT if additional diagnostic evaluations, such as PSMA PET scan, rule out the presence of extraprostatic disease (Expert Opinion). The consensus statements regarding the use of PSA velocity and density are variable. These markers were incorporated into the initial consensus statements for FT [6]. Additionally, the potential role of PSA density in this context has been recently endorsed by Marra. et al. [15]. However, Tay et al. did not reach a consensus on this matter [10]. Other PSA-related markers, such as prostate health index (PHI) and 4k score, have demonstrated a higher accuracy in identifying clinically significant disease compared to PSA alone [50, 51]. However, their role in patient selection for FT remains unknown. In a recent Delphi consensus by Marra et al. 80% of participants agreed that that evidence for molecular biomarkers in FT is absent or low. Hence, the panel did not endorse their utilization in routine clinical decision-making [15]. Nevertheless, some experts believe that these biomarkers may play a role in the process of patient selection for FT with localizing occult clinically significant PCa and quantifying the potential risk of cancer progression.

## Future directions

There is increasing data on the use of genetic testing in the management of all stages of PCa; however, there have been no definitive studies on the utility of germ line or somatic genetic testing in evaluating patients for FT. The earliest data that is similar to men when considering FT concerns genetic testing and long-term outcomes in PCa and in when deciding on active surveillance.

Men who have a BRCA 1/2 or ATM mutation with newly diagnosed PCa are more likely to have aggressive disease and die from PCa [52]. In studies of genetic mutation on men considering active surveillance these same mutations are associated with upgrading and progression in men on active surveillance [53]. While not directly addressing decision making in FT, men with these germ line mutations do not appear to be ideal candidates for FT due to the high likelihood of progression and death from the disease.

In recent years, several serum, urine, and tissue-based genetic and epigenetic biomarkers have emerged as novel risk-stratification tools for PCa. Some of the tests using these biomarkers include:

- Prolaris: It is a tissue-based quantitative reverse transcription polymerase chain reaction (RT-PCR) test that measures mRNA levels of 31 cell cycle progression and 15 housekeeping genes. This test provides an independent prediction of PCa-specific mortality and is available for use in men diagnosed with very low and low-risk PCa [54, 55].
- OncotypeDX: It is a tissue-based quantitative RT-PCR test that measures the expression 12 cancer-specific and 5 housekeeping genes. It predicts favorable vs. adverse pathology (defined as Gleason ≥ 4 + 3 or ≥ pT3). The results are presented as genomic prostate score (GPS). For every 20-point increase in the GPS, there is a twofold elevation in the risk of adverse pathology observed at radical prostatectomy. This test is currently validated for men with low- to low-intermediate risk disease considering active surveillance [56–58].
- Decipher: It is a tissue-based gene expression classifier which is designed based on the microarray expression of 22 genomic marker signatures. This broadly validated test provides several prognostic variables, including the risk of adverse pathology, percentage risk of distant metastasis in 5- and 10-years, and 15-year disease specific mortality [59–62].

Despite the promising role of these biomarkers in selecting patients for biopsy, active surveillance, and definitive therapy, which is endorsed by current guidelines [5, 48], their utility and efficacy in the field of FT has yet to be determined.

## Conclusion

During the past two decades, there has been a gradual shift in FT towards targeting larger volumes and higher grades of PCa moving away from primarily addressing low-volume and low-grade tumors. Currently, FT is considered in well-selected patients with intermediate-risk PCa. Precise patient selection is a fundamental step in achieving the optimal functional and oncological outcomes in these patients. Current selection criteria are based on the international multidisciplinary consensus statements. The ongoing trials and studies investigating novel genomic and molecular biomarkers will provide a higher level of evidence in this setting and shed light on the optimal patient selection criteria for FT.

## Data Availability

Data supporting the findings of this study are available within the article.
